# A rare *KMT2A::CBL* transcript in an acute monoblastic leukemia patient with an unfavorable outcome

**DOI:** 10.1007/s11033-024-09543-0

**Published:** 2024-04-21

**Authors:** Jinglei Yu, Fengmei Song, Mingming Zhang, Pingnan Xiao, Jingjing Feng, Ruimin Hong, Yongxian Hu, He Huang, Guoqing Wei

**Affiliations:** 1 Bone Marrow Transplantation Center, The First Affiliated Hospital, Zhejiang University School of Medicine, Hangzhou, China; 2 Liangzhu Laboratory, Zhejiang University Medical Center, Hangzhou, China; 3 Institute of Hematology, Zhejiang University, Hangzhou, China; 4 Zhejiang Province Engineering Laboratory for Stem Cell and Immunity Therapy, Hangzhou, China

**Keywords:** KMT2A, CBL, Acute monoblastic leukemia, Acute myeloid leukemia

## Abstract

**Background:**

Lysine [K] methyltransferase 2A (*KMT2A*, previously known as *MLL*) gene rearrangements are common in acute leukemias of various lineages and are associated with features such as chemotherapy resistance and rapid relapse. *KMT2A::CBL* is a rare fusion of unknown pathogenesis generated by a unique interstitial deletion of chromosome 11 that has been reported across a wide age range in both acute myeloid leukemia (AML) and acute lymphoblastic leukemia (ALL) patients. The leukemogenic effect of the *KMT2A::CBL* rearrangement and its association with clinical prognosis have not been well clarified.

**Methods and results:**

We report the case of a 64-year-old female who was diagnosed with acute monoblastic leukemia (M5a) and who acquired the rare *KMT2A::CBL* fusion. The patient received multiple cycles of therapy but did not achieve remission and eventually succumbed to severe infection and disease progression. Additionally, we characterized the predicted KMT2A-CBL protein structure in this case to reveal the underlying leukemogenic mechanisms and summarized reported cases of hematological malignancies with *KMT2A::CBL* fusion to investigate the correlation of gene rearrangements with clinical outcomes.

**Conclusions:**

This report provides novel insights into the leukemogenic potential of the *KMT2A::CBL* rearrangement and the correlation between gene rearrangements and clinical outcomes.

**Supplementary Information:**

The online version contains supplementary material available at 10.1007/s11033-024-09543-0.

## Introduction

Lysine [K] methyltransferase 2A (*KMT2A*, previously known as *MLL*) gene rearrangements are common in various subtypes of acute leukemia and are related to chemotherapy refractoriness and adverse prognosis [1]. Commonly involved in acute leukemias of myeloid, lymphoid, and mixed lineages, *KMT2A* rearrangements encode KMT2A fused with a highly diverse range of partner genes [2]. As one of the *KMT2A* fusion partners, *CBL* is a proto-oncogene telomeric to *KMT2A* at 11q23.3 with a length of 680 kb. It encodes a multifunctional protein that can negatively modulate tyrosine kinase-related signaling as a kind of ubiquitin ligase (E3) or positively transduce signals as a multidomain adaptor protein [3].

*KMT2A::CBL* fusion is believed to result from interstitial cryptic loss of the 3’*KMT2A* signal rather than from specific translocation between two homologous forms of chromosome 11 [4]. Although uncommon, *KMT2A::CBL* fusion has been reported in both pediatric and adult acute leukemias, including AML and ALL [5]. Nevertheless, the precise role of *KMT2A::CBL* fusion in leukemogenesis and the correlation of this unique recombination with clinical outcomes remain unknown.

**Fig. 1:**
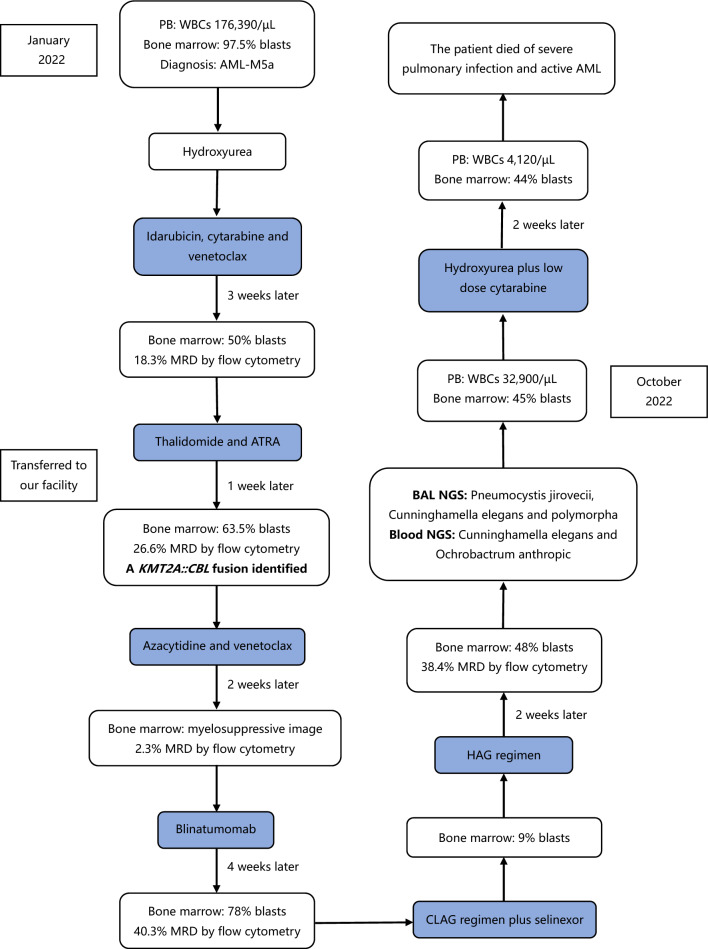
Treatment course

**Fig. 2:**
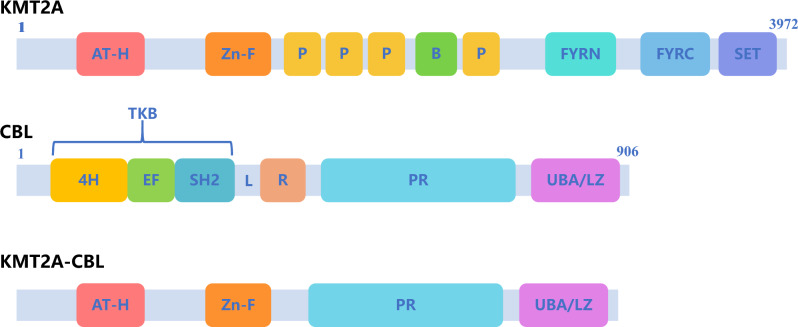
Schematic representation of normal KMT2A and CBL and the predicted KMT2A-CBL fusion proteins. The predicted KMT2A-CBL fusion protein retained the AT hooks and CXXC zinc finger domain of KMT2A, as well as the proline-rich domain and UBA/LZ region of CBL. AT-H AT hooks; Zn-F CXXC zinc finger domain; P plant homeotic domain; B bromodomain; FYRN F/Y-rich N-terminus domain; FYRC F/Y-rich C-terminus domain; SET Su(var)3–9, enhancer of Zeste, trithorax domain; 4H 4-helix bundle; EF EF hand calcium-binding; SH2 Src homology 2; TKB tyrosine kinase-binding domain; L linker region; R RING finger domain; PR proline-rich domain; UBA/LZ ubiquitin-associated/leucine zipper domain

We describe a patient with acute monoblastic leukemia (M5a) harboring the rare *KMT2A::CBL* fusion who was refractory to multiple lines of therapy and eventually died from severe infection and disease progression (Fig. 1). In addition, we characterized the predicted KMT2A-CBL protein structure in this patient (Fig. 2) to enhance the understanding of the underlying leukemogenic mechanisms and summarized reported cases of hematological malignancies with *KMT2A::CBL* fusion to investigate the correlation of gene rearrangements with clinical outcomes.

## Case presentation

A 64-year-old female diagnosed with acute monoblastic leukemia (M5a) was transferred to our hospital in February 2022 in the wake of nonremission and a febrile state after induction chemotherapy at a local hospital. At baseline, analysis of her peripheral blood (PB) revealed markedly increased white blood cells (WBCs; 176,390/µL), moderate anemia (hemoglobin; 8.2 g/dL), and thrombocytopaenia (platelets; 80,000/µL). Initial bone marrow analysis revealed hypercellularity, with 97.5% of cells being monoblasts and promonocytes expressing CD34, HLA-DR, CD117, CD33 (dim), CD13, and CD15 (small subset). Cytogenetic analysis revealed an aberrant karyotype: 46,XX,del(7)(q22)[18]/46,XX[2]. After diagnosis, the patient was immediately started on idarubicin, cytarabine, and venetoclax therapy (because of infection, the venetoclax treatment was stopped midway), followed by thalidomide and all-trans retinoic acid (ATRA) treatment three weeks later because reevaluation of the bone marrow aspirate showed 50% blasts by morphology and 18.3% myeloid blast cells by flow cytometry.

**Fig. 3:**
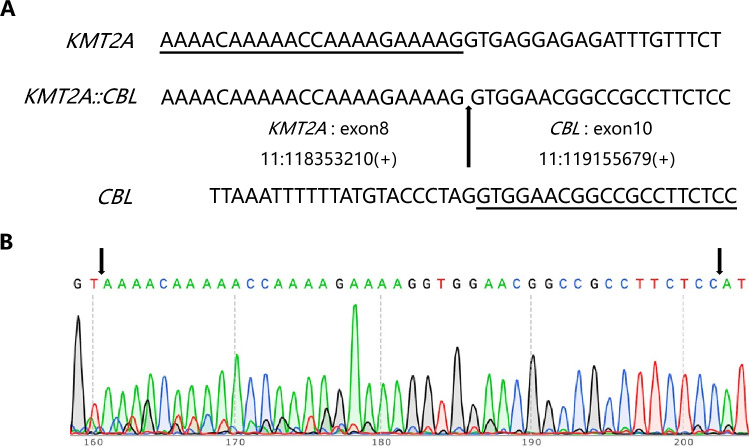
Genomic analysis of the *KMT2A::CBL* fusion transcript. **A** cDNA sequence of the *KMT2A::CBL* breakpoint region determined by RNA-based NGS, and the normal sequence fragments of *KMT2A* and *CBL* were used as controls. The arrow indicates the breakpoint. The fusion sequences are underlined. **B** The sequence of the *KMT2A::CBL* breakpoint region was validated by RT-PCR with Sanger sequencing. The arrows in the peak diagram indicate the verified sequence

**Fig. 4:**
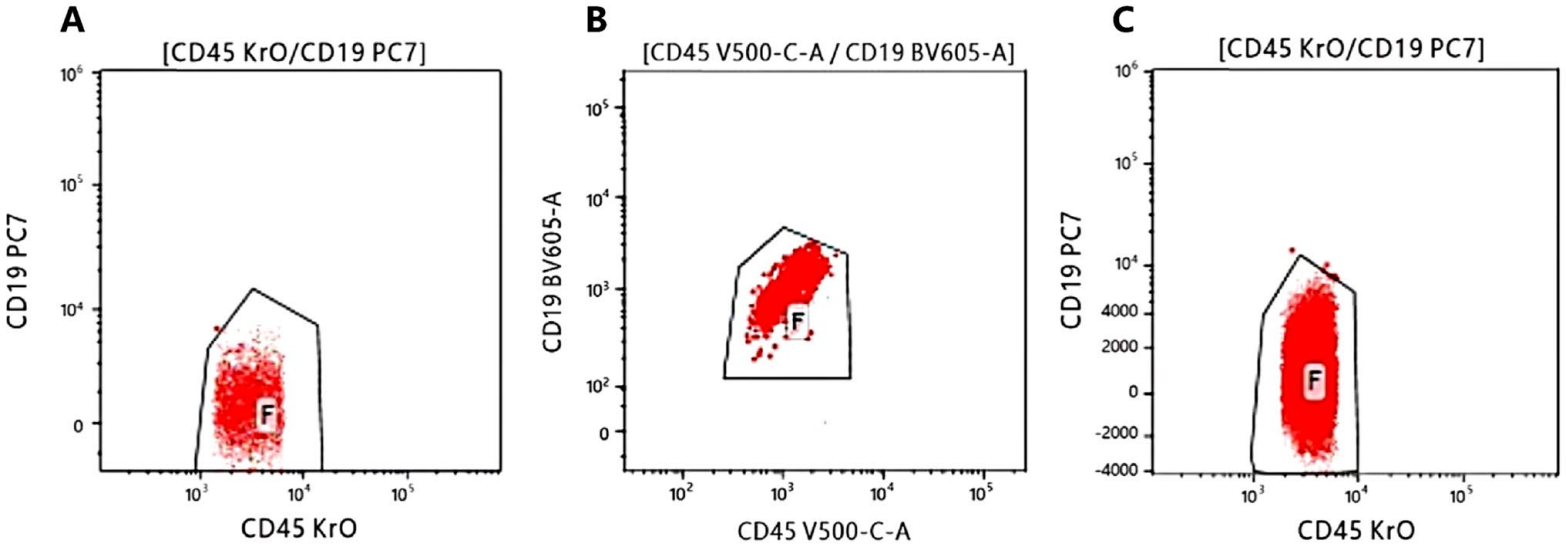
The immunophenotypic shift in CD19 surface expression. **A** The initial expression of CD19 in the patient upon admission. **B** The leukemia cells of the patient showed an immunophenotypic shift in CD19 after the third cycle of myeloid-directed therapy. **C** CD19 surface expression was unexpectedly lost after CD19-targeted immunotherapy

One month after initial diagnosis, the patient had 63.5% blasts with monocytic differentiation according to bone marrow smears (Figure S1A). The surface expression of CD117, CD34, CD13, CD33, HLA-DR, CD123, CD15, and CD38 (dim) was determined. Moreover, a *KMT2A::CBL* fusion transcript was identified via RNA-based next-generation sequencing (NGS) through alignment to the reference genome and fusion detection algorithms, which was validated via RT-PCR combined with Sanger sequencing (Fig. 3). Because of her unstable clinical condition, the patient was started on a nonintensive regimen of azacytidine and venetoclax. However, two weeks after therapy, a bone marrow cytologic study revealed myelosuppression with a decrease in the number of nucleated cells, and flow cytometry revealed that the abnormal cells exhibited loss of CD13 and CD33 expression but increased CD19 and CD7 expression (Fig. 4B). At that time, the patient suffered from severe neutropenia, thrombocytopenia and anemia, and did not achieve remission by the end of this treatment cycle. As a consequence, the patient was diagnosed with primary refractory AML.

Afterward, blinatumomab was administered because the leukemia cells were CD19 positive. However, the bone marrow smear showed 78% myeloblasts with fine granular chromatin and small prominent nucleoli (Figure S1B) one month later, and CD19 was negative (Fig. 4C). For that reason, the patient received CLAG (cladribine, cytarabine, and G-CSF) combined with selinexor as salvage chemotherapy and HAG (homoharringtonine, cytarabine, and G-CSF) as maintenance therapy. Unfortunately, her marrow smears later still showed 48% myeloblasts and minimal residual disease (MRD) positivity. Her cytogenetic analysis revealed novel karyotypes (45,XX,del(7)(q22q34),-15[9]/46,XX[1]), heralding disease progression. Moreover, the patient developed a high fever, and her lung computed tomography scan showed progression of pulmonary infection. Analysis of her bronchoalveolar lavage (BAL) revealed infection with *Pneumocystis jirovecii*, *Cunninghamella elegans* and *Cunninghamella polymorpha* by gene sequencing. Her peripheral blood test also revealed the emergence of circulating blasts and increasing WBC counts. Although aggressive treatment was implemented, the patient’s condition rapidly deteriorated, eventually leading to severe sepsis and death.

## Discussion

*KMT2A* and *CBL* are located between the STS markers *D11S939* and *D11S924* on the 11q23.3 chromosomal segment [6]. *CBL* was the second gene found to be fused with *KMT2A* through interstitial deletion, followed by the *LARG* gene [6]. The *KMT2A::CBL* fusion transcript in our patient contained *KMT2A* exon 8 fused to *CBL* exon 10 (Fig. 3). Accordingly, the predicted KMT2A-CBL fusion protein retained the AT hooks and CXXC zinc finger domain of KMT2A [7], as well as the proline-rich domain and UBA/LZ region of CBL [8] (Fig. 2), suggesting a novel fusion.

*KMT2A* rearrangement is regarded as an indicator of poor prognosis with unique involvement in early leukemogenesis [9]. Mutations in the *CBL* gene are frequently detected in myeloid neoplasms and are associated with pathogenesis onset [3]. However, the leukemogenic effect of the *KMT2A::CBL* fusion remains to be revealed.

We reviewed the other 7 published cases and generalized the characteristics of hematologic malignancies harboring the *KMT2A::CBL* fusion together with our case; 4 of the patients had ALL, and 4 had AML (Table S1) [1, 6, 10, 11]. Notably, outcomes were relatively worse among the AML patients, with 50% of patients dying and 25% experiencing relapse. To our knowledge, another 10 cases of hematologic neoplasms harboring a *KMT2A::CBL* fusion have been reported recently and are not included in this table [5]. It is worth further summarizing these findings to improve the knowledge about the related clinical outcomes.

The patient in our case presented with primary refractory AML with high-risk clinical features. After the third cycle of myeloid-directed therapy, an interesting immunophenotypic shift was observed in which her leukemia cells exhibited increased CD19 and CD7 surface expression but decreased CD13 and CD33 expression (Fig. 4B). Notably, CD19 surface expression was unexpectedly lost after CD19-targeted immunotherapy (Fig. 4C). These findings may be partially explained by the lineage shift that can occur under selective pressure in *KMT2A*-rearranged acute leukemias, which is possibly associated with disease progression [12]. Primitive pluripotent progenitors, capable of differentiating into cells of an alternative lineage, may be the basis of this kind of therapy-mediated selection [12]. Furthermore, *KMT2A* fusions remain a major adverse prognostic factor in mixed-phenotype acute leukemia (MPAL) [13]. Thus, the poor clinical outcome of our patient was largely attributed to the development of the *KMT2A::CBL* fusion.

Although *KMT2A* rearrangement has been identified as a high-risk marker in both ALL and AML, it is believed that the specific fusion partner plays an indispensable role in pathogenesis and can influence the outcome to some extent [14, 15]. CBL has four primary functional domains, is localized in the cytoplasm and is highly expressed in hematopoietic cells [3]. The amino-terminal region of CBL contains a tyrosine kinase-binding (TKB) domain, which is composed of a four-helix (4H) bundle, a Ca^2+^-binding EF (EF) hand, and a Src homology 2 (SH2) region that binds to the RING finger through a short linker (L) domain. The RING finger domain is predicted to recruit an E2 ubiquitin-conjugating enzyme and degrade the tyrosine kinase linked to the TKB domain, thus negatively regulating signaling [3, 6]. Interestingly, the *CBL* mutant is thought to disrupt the original E3 ubiquitin ligase activity and promote cancer onset and progression [3]. When CBL truncation causes loss of the TKB or RING finger domain, the resulting protein will likely confer a gain of oncogenic function, which may partially explain the leukemogenic mechanism of *KMT2A::CBL* rearrangement in our case.

In addition to the transforming effect of the truncated CBL protein, dimerization of the KMT2A-CBL protein may be another leukemogenic mechanism of the *KMT2A::CBL* fusion. The carboxy-terminal region of CBL is a conserved sequence termed the ubiquitin-associated (UBA) domain, which also contains a leucine zipper (LZ) domain capable of mediating homooligomerization. Coincidentally, dimerization of the chimeric KMT2A protein, which is mediated by the LZ domain of fusion partners, is reportedly necessary and sufficient for leukemogenesis [6]. In conclusion, the chimeric protein has a novel function in the abnormal cellular setting that contributes to pathogenesis [1].

Acute leukemias with *KMT2A::CBL* rearrangements have not been reported in many studies. The correlation between this gene rearrangement and clinical outcome has not been clearly explained. However, based on a review of the existing reports, AML patients with *KMT2A::CBL* rearrangements usually have a dismal clinical outcome. It is also important to note that infections that occur in patients with AML due to immunodeficiency caused by chemotherapy are not only challenging to treat but also may cause deterioration of the patient’s condition or even death, as shown in our case. For *KMT2A::CBL* rearrangements, there is a lack of risk stratification strategies and targeted precision treatments. An enhanced comprehension of the underlying leukemogenic mechanisms of *KMT2A::CBL* fusions has important implications for better understanding the disease and will provide insights into the molecular events involved in leukemogenesis and ideas for appropriate treatments.

## Conclusions

In conclusion, we present a case of *KMT2A::CBL*-positive acute monoblastic leukemia with a dismal prognosis. Based on previous studies, *KMT2A::CBL* fusion can be considered a rare high-risk fusion with aggressive leukemogenic effects. Further studies are needed to identify the characteristics of *KMT2A::CBL* rearrangements and explore appropriate treatment modalities.

## Supplementary Information

Below is the link to the electronic supplementary material.

Supplementary file1 (DOCX 2447 KB)

## Data Availability

All data that support the findings of this study are included in this article. Further enquiries can be directed to the corresponding author.

## Abbreviations

AML: Acute myeloid leukemia
ALL: Acute lymphoblastic leukemia
M5a: Acute monoblastic leukemia
PB: Peripheral blood
WBCs: White blood cells
ATRA: All-trans retinoic acid
MRD: Minimal residual disease
BAL: Bronchoalveolar lavage
sAML: Secondary acute myeloid leukemia
NGS: Next-generation sequencing
CR: Complete response
FAB: French-American-British classification
EGIL: European Group for the Immunologic Classification of Leukemia
MUD: Matched unrelated donor
RD: Related donor
CB: Cord blood
AlloSCT: Allogeneic stem cell transplantation
VAF: Variant allele frequency
MPAL: Mixed-phenotype acute leukemia
RT-PCR: Reverse transcription polymerase chain reaction
