# Supplementary material for: A rare KMT2A::CBL transcript in an acute monoblastic leukemia patient with an unfavorable outcome
Source: Mol Biol Rep. 2024 Apr 21;51(1):561. doi: 10.1007/s11033-024-09543-0 (PMC11033236; doi:10.1007/s11033-024-09543-0)

**A rare *KMT2A::CBL* transcript in an acute monoblastic leukemia patient with an unfavorable outcome**

*Molecular Biology Reports*

Jinglei Yu^1,2,3,4^, Fengmei Song^1,2,3,4^, Mingming Zhang^1,2,3,4^, Pingnan Xiao^1,2,3,4^, Jingjing Feng^1,2,3,4^, Ruimin Hong^1,2,3,4^, Yongxian Hu^1,2,3,4^, He Huang^1,2,3,4^ and Guoqing Wei^1,2,3,4,*^

*^1^Bone Marrow Transplantation Center, The First Affiliated Hospital, Zhejiang University School of Medicine, Hangzhou, China*

*^2^Liangzhu Laboratory, Zhejiang University Medical Center, Hangzhou, China*

*^3^Institute of Hematology, Zhejiang University, Hangzhou, China*

*^4^Zhejiang Province Engineering Laboratory for Stem Cell and Immunity Therapy, Hangzhou, China*

*Corresponding Author:

Guoqing Wei

Bone Marrow Transplantation Center, The First Affiliated Hospital of Zhejiang University School of Medicine

No.79 Qingchun Road, Hangzhou, China

Tel: +86-13867475373, E-mail: [weiguoqing2018@zju.edu.cn](mailto:weiguoqing2018@zju.edu.cn)

**Acknowledgments**

The authors thank American Journal Experts (AJE) for English language editing of the manuscript.

Table S1. Patients with *KMT2A::CBL* rearrangement.

| Reference [Year] | Sex, age(y) | Hematologic diagnosis | No. of CRs | Immunology | Cytogenetics | *KMT2A* FISH | NGS | Breakpoint | Fusion transcript | Outcome |
| --- | --- | --- | --- | --- | --- | --- | --- | --- | --- | --- |
| AML | | | | | | | | | | |
| Fu et al. [2003] | Female, 28 | AML (FAB M1) | Three | CD34^+^, CD13^+^, CD33^+^ | 50,XX,+22,+3mar[5]/49,XX,-19,+22,+3mar[11]/46,XX,inv(9)[4] | - | *CBL* p.L380P (VAF 70.0%) | *KMT2A*: intron 6  *CBL*: intron 7 | *KMT2A*: exon 6~*CBL*: exon 8 | CR achieved; proceeded to MUD-AlloSCT; however, died of AlloSCT complications |
| Meyer et al. [2018] | Female, 59 | sAML (FAB M5) | Two | CD34^-^, CD13^+^, CD33^+^, CD4^+^ | 45~46,XX,-5,add(6)(p21),-9,+i(9)(p10),add(11)(q23),-18,+mar[7]/47~49,XX,+2,-5,-9,i(9q10),add(11)(q23),?+13,-18,+19,+20,inc[9] | deletion of *KMT2A* 3’portion | - | *KMT2A*: intron 9  *CBL*: intron 9 | *KMT2A*: exon 9~*CBL*: exon 10 | CR achieved; proceeded to RD-AlloCST; however, relapse 11months after diagnosis |
| Boneva et al. [2020] | Female, 18 | AML | One | CD34^+^, cMPO^+^, CD33^+^, CD15^+^, CD38^+^, CD56^+^ | 46,XX[20] | deletion of *KMT2A* 3’portion | *RUNX1* p.G372S (VAF 50.0%) | *KMT2A*: intron 10  *CBL*: exon 2 | *KMT2A*: exon 10~*CBL*: exon 2 | CR achieved; loss of follow-up |
| Current [2023] | Female, 64 | AML (FAB M5a) | None | Initial report: CD34^+^, CD117^+^, HLA-DR^+^, CD13^+^, CD33^+^dim, CD15^+^, CD38^-^, CD56^-^, CD7^-^, CD19^-^, CD20^-^;  2 months later: CD34^-^, CD117^+^, HLA-DR^+^, CD13^-^, CD33^-^, CD7^+^, CD19^+^, CD10^-^, CD45^+^dim;  4 months later: CD34^-^, CD117^+^, HLA-DR^+^, CD13^-^, CD33^-^, CD7^-^, CD19^-^, CD10^-^, CD45^+^dim, CD38^+^; | Initial report: 46,XX,del(7)(q22)[18]/46,XX[2]  4 months later: 45,XX,del(7)(q22q34),-15[9]/46,XX[1] | - | *TET2* p.H1077Tfs*5 (VAF 23.9%)  *TET2* p.S716* (VAF 0.8%)  *TET2* p.H1868Y (VAF 29.3%)  *RAD21* p.L567Sfs*7 (VAF 22.7%)  *SRSF2* p.P95L (VAF 25.0%) | - | *KMT2A*: exon 8~*CBL*: exon 10 | Persistent disease; died of severe pulmonary infection and disease progression 10 months after diagnosis |
| ALL | | | | | | | | | | |
| Meyer et al. [2018] | Male, 3 | B-ALL (EGIL B1) | Three | CD34^-^, TdT^+^, CD19^+^, CD79a^+^, CD10^+^, CD7^+^dim, CD38^+^ | - | deletion of *KMT2A* 3’portion | *KRAS* p.A18D (VAF 44.0%) | *KMT2A*: intron 10  *CBL*: intron 9 | *KMT2A*: exon 10~*CBL*: exon 10 | CR achieved; alive at CR 13 years after diagnosis |
| Alex et al. [2021] | Male, 9 | T-ALL (EGIL T2) | Two | cCD3^+^, sCD3^-^, TdT^+^, CD99^+^, CD7^+^, CD2^+^, CD5^+^ | Pseudodiploid and monosomic with 3 clonal anomalies: del(2q),-16,r | deletion of *KMT2A* 3’portion | *KRAS* p.G13C (VAF 32.0%)  *ASXL1* p.G646Wfs12 (VAF 26.0%)  *STAT3* p.N481l (VAF 36.0%) | *KMT2A*: intron 9  *CBL*: intron 9 | *KMT2A*: exon 9~*CBL*: exon 10 | CR achieved; proceeded to CB-AlloSCT; alive at CR 1 year after diagnosis |
| Alex et al. [2021] | Male, 11 | T-ALL (EGIL T2) | One | cCD3^+^, sCD3^-^, CD7^++^, CD2^++^, CD5^+/-^(13%), CD34^+^dim, CD38^++^, CD13^+^ | 47,XY,?del(5)(q31),+8,del(9)(q13q34),del(11)(q21q23),del(12)(p11)[cp6]/46,XY[28] | deletion of *KMT2A* 3’portion | *IL7R* p.V253_A254insVA (VAF 13.0%)  *KMT2A* p.N*2278fs (VAF 82.0%)  *NOTCH1* p.P2514fs (VAF 16.0%)  *NOTCH1* p.A1700D (VAF 10.0%)  *NOTCH1* p.L1678P (VAF 36.0%)  *ZFHX4* p.R2676Q (VAF 28.0%) | *KMT2A*: exon 10  *CBL*: intron 15 | *KMT2A*: exon 10~*CBL*: exon 16 | CR achieved; alive at CR 3 years after diagnosis |
| Alex et al. [2021] | Male, 18 | T-ALL (EGIL T2) | Two | cCD3^+^, sCD3^+^dim, CD34^-^, TdT^-^, CD99^+^dim, CD1a^-^ CD7^++^, CD38^+^, | 47,XY,der(7),+19[20] | deletion of *KMT2A* 3’portion | No mutations detected | *KMT2A*: intron 9  *CBL*: intron 9 | *KMT2A*: exon 9~*CBL*: exon 10 | CR achieved; proceeded to MUD-AlloSCT; alive at CR 6 months after diagnosis |

**Abbreviations:** sAML, secondary acute myeloid leukemia; NGS, next-generation sequencing; CR, complete response; FAB, French-American-British classification; EGIL, European Group for the Immunologic Classification of Leukemia; MUD, matched unrelated donor; RD, related donor; CB, cord blood; AlloSCT, allogeneic stem cell transplantation; VAF, variant allele frequency.

Figure S1. Cytology of bone marrow cells. (A) Bone marrow smears showed heavy infiltration of large cells with folded nuclei, fine chromatin, and large prominent nucleoli. The blast percentage was 63.5%. (B) The bone marrow smear showed 78% myeloblasts with fine granular chromatin and small prominent nucleoli.

A


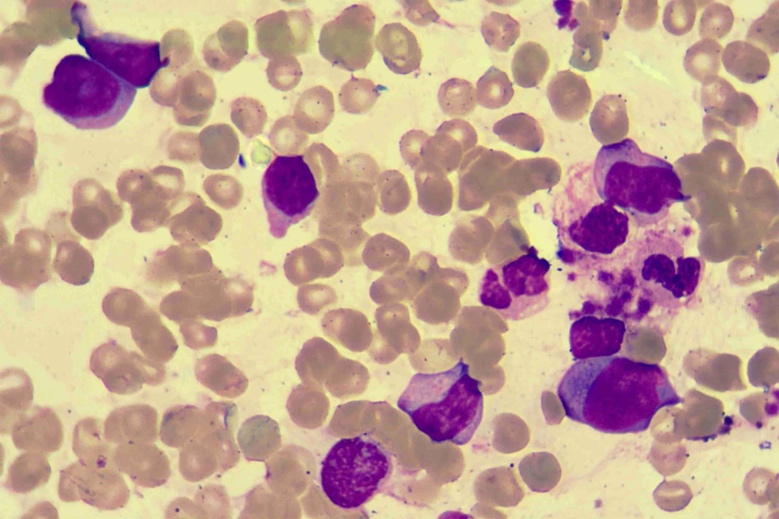


B


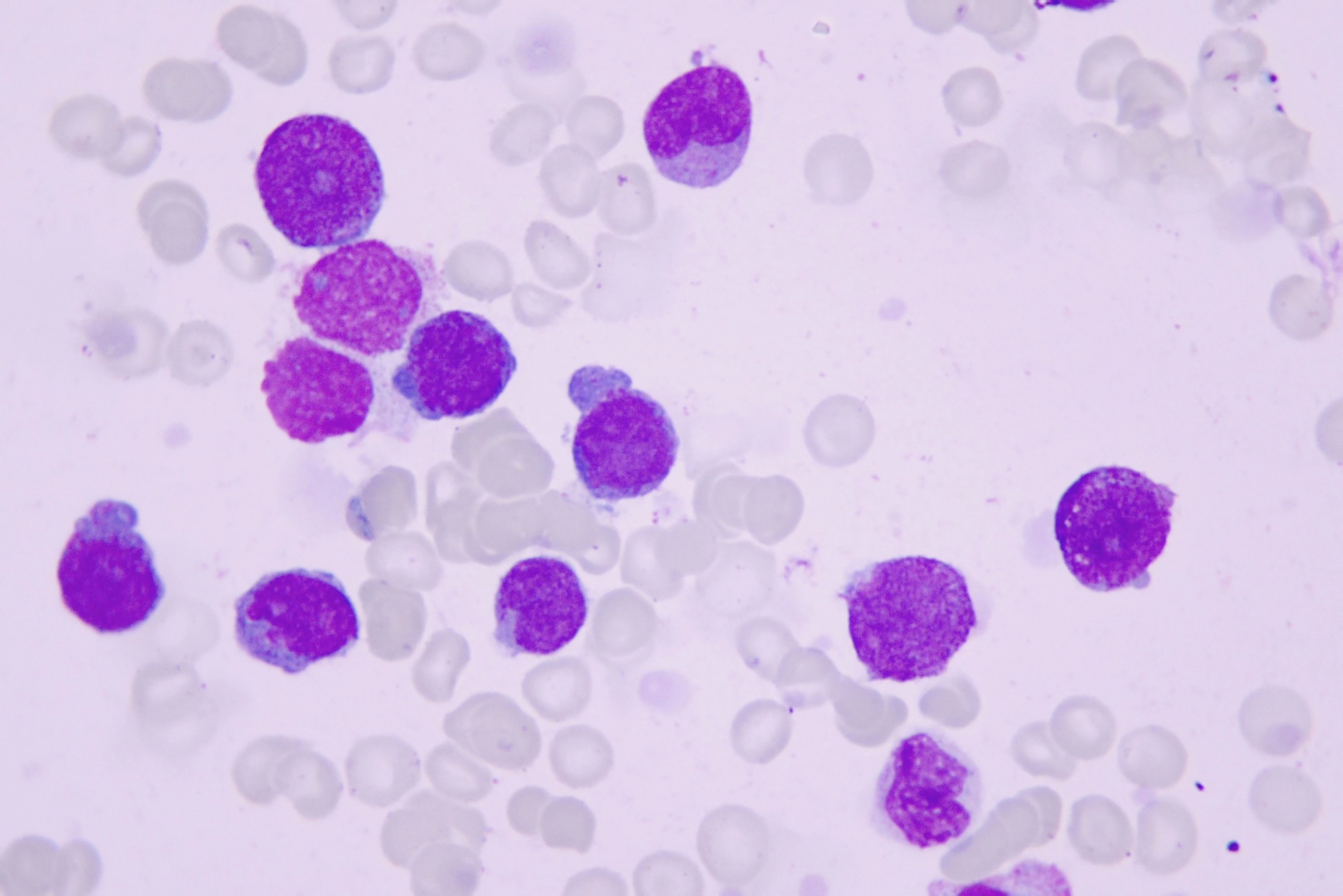

Supplement: Supplementary file 1 — Supplementary file1 (DOCX 2447 KB) [file 11033_2024_9543_MOESM1_ESM.docx]
